# Antimicrobial resistance in colonizing group B *Streptococcus* among pregnant women from a hospital in Vietnam

**DOI:** 10.1038/s41598-021-00468-3

**Published:** 2021-10-21

**Authors:** Vu Van Du, Pham Thai Dung, Nguyen Linh Toan, Can Van Mao, Nguyen Thanh Bac, Hoang Van Tong, Ho Anh Son, Nghiem Duc Thuan, Nguyen Thanh Viet

**Affiliations:** 1 National Hospital of Obstetrics and Gynecology, Hanoi, 100000 Vietnam; 2 Intensive Care Unit, 103 Military Hospital, Hanoi, 100000 Vietnam; 3 Department Post-Graduate Training Management, Vietnam Military Medical University (VMMU), Hanoi, 100000 Vietnam; 4 Department of Pathophysiology, VMMU, Hanoi, 100000 Vietnam; 5 Department of Neurosurgery, 103 Military Hospital, Hanoi, 100000 Vietnam; 6 Institute of Biomedicine and Pharmacy, VMMU, Hanoi, 100000 Vietnam; 7 Vietnam Military Medical University (VMMU), Hanoi, 100000 Vietnam

**Keywords:** Bacterial pathogenesis, Infectious-disease epidemiology, Antimicrobials, Bacteria, Pathogens, Bacterial infection, Infectious diseases, Diseases, Medical research, Pathogenesis, Risk factors

## Abstract

Few studies have been conducted on group B *Streptococcus* (GBS) in Vietnam. We determined the GBS colonization and antimicrobial resistance vaginal-rectal profile of 3863 Vietnamese pregnant women over 5 years. Maternal GBS colonization was characterized by antibiotic susceptibility. Overall, the GBS colonization rate was 8.02% (95% CI: 7.20–8.94%). Compared to sampling ≥ 35 weeks of gestation, the GBS colonization rate was statistically higher (*p* = 0.004) with sampling < 35 weeks. Among 272 antimicrobial susceptibility testing isolates, all were susceptible to ampicillin, penicillin, ceftriaxone, cefotaxime, vancomycin, and quinupristin/dalfopristin. Resistance was highest for tetracycline (89.66%), followed by erythromycin (76.23%) and clindamycin (58.21%). Multidrug resistance and resistance to ≥ 6 different antibiotics were 60.66% and 8.82%, respectively. Resistance to clindamycin but not erythromycin (L phenotype) was 2.2%. The clindamycin resistance rate was significantly increased (*p* = 0.005) during the study period. These data demonstrate a low rate of maternal GBS colonization. The high rate of erythromycin, clindamycin, and multidrug resistance to GBS that can be transmitted to neonates is an important risk factor to consider. β-lactams continue to be appropriate for first-line treatment and prophylaxis in the study area. Ongoing monitoring should be considered in the future.

## Introduction

Group B *Streptococcus* (GBS) causes severe early-onset infection in newborns. GBS is a Gram-positive, catalase-negative coccus found in pairs and chains on Gram staining^1^. GBS causes neonatal mortality and significant morbidity, including meningitis and sepsis^2^. Maternal GBS colonization relates to preterm birth, pregnancy loss^3^, and stillbirths^4^. GBS colonization among pregnant women has an average of 18%, ranging from 11 to 35%, totalling 21.7 million in 195 countries^5^. Maternal GBS colonization can be transmitted to neonates before or during birth^6^. The maternal GBS colonization rates vary geographically^5^. The Centers for Disease Control and Prevention (CDC) recommends screening for GBS colonization in all pregnant women to prevent newborn infection^7^. GBS monitoring provides information for decision-making, planning, prevention, and control strategies^8–10^.

Antibiotics are a vital resource to treat bacterial infections. Thus, antibiotic-resistant bacteria are a public health concern. The reduced susceptibility rate to penicillin and ampicillin, the first-choice drugs for the prevention and treatment of maternal and neonate GBS infections, is increasing^11–14^. Erythromycin and clindamycin are the second-choice drugs, particularly in cases of penicillin allergy^7^. However, resistance to such antibiotics has increased in recent years, raising concerns about their use as alternatives^11,13,14^. Resistance to other classes of antibiotics continues to increase, making it necessary to monitor antimicrobial resistance globally^15^. Resistance data could (1) provide evidence to improve clinical practice; (2) help to develop antimicrobial-prescribing guidelines; and (3) aid prospective interventions (such as antimicrobial prophylaxis)^16^.

Maternal GBS colonization and antibiotic resistance profiles vary geographically^8^. Few studies have been conducted on GBS in Vietnam. To date, only one study reported that the maternal GBS colonization rate in a province of Vietnam was 9.2%^17^. To contribute to regional data on maternal GBS colonization and antibiotic resistance profiles, an observational study of Vietnamese pregnant women was conducted at a hospital in Hanoi, Vietnam. These findings may provide information to guide disease prevention practices in developing countries. In Vietnam, the infrequent use of intrapartum antibiotic prophylaxis (IAP) and the underestimation of neonatal morbidity and mortality are of concern.

## Results

### GBS colonization and maternal GBS colonization characteristics

Table 1 provides the baseline characteristics of maternal GBS colonization. A total of 3,863 pregnant women participated in this study. The rate of GBS colonization was 8.02% (95% CI: 7.20–8.94%). The rates of GBS colonization of pregnant women at ≥ 35 weeks and < 35 weeks of gestational age were 3.34% (95% CI: 2.82–3.95%) and 4.66% (95% CI: 4.04–5.37%), respectively. The rate of maternal GBS colonization sampled at < 35 weeks was significantly higher than that sampled at ≥ 35 weeks gestation (*p* = 0.004).

**Table 1:** The baseline characteristics of maternal GBS colonization.

Characteristics	Total (n = 310)	Weeks of gestation, n (%)**		*p* value
	n (%)	≥ 35 (n = 129)	< 35 (n = 180)	
**Clinical and obstetrics**				
Total number of positive isolates	310 (8.02)*	129 (3.34)*	180 (4.66)*	0.004
Maternal mean age (range)	30.49 (18–54)	31.14 (18–49)	30.03 (18–54)	0.150^b^
Mean gestational age (range)	31.32 (8–41.2)	38.28 (35–41.2)	26.33 (8–34.6)	< 0.001^b^
Preterm birth	6 (1.94)	0 (0)	6 (3.33)	0.043^a^
Stillbirth	12 (3.87)	1 (0.78)	11 (6.11)	0.036^a^
Primiparous	129 (41.61)	71 (55.04)	58 (32.22)	< 0.001
2nd Parity	62 (20)	22 (17.05)	40 (22.22)	0.329
3rd Parity	30 (9.68)**	12 (9.3)	17 (9.44)	1
4th Parity	10 (3.23)	3 (2.33)	7 (3.89)	0.530^a^
5th Parity	1 (0.32)	1 (0.78)	0 (0)	0.420^a^
Parity (not available)	78 (25.16)	20 (15.5)	58 (32.22)	0.001
In vitro fertilization (IVF)	55 (17.74)	8 (6.2)	47 (81.67)	< 0.001
**Occupation**				
Self-employed	103 (33.23)**	43 (33.33)	59 (32.78)	1
Office worker	77 (24.84)	37 (28.68)	40 (22.22)	0.245
Teacher	62 (20)	24 (18.6)	38 (21.11)	0.69
Factory worker	31 (10)	11 (8.53)	20 (11.11)	0.579
Farmer	24 (7.74)	13 (10.08)	11 (6.11)	0.285
Student	5 (1.61)	0 (0)	5 (2.78)	0.077^a^
Health care worker	4 (1.29)	1 (0.78)	3 (1.67)	0.643^a^
Engineer	2 (0.65)	0 (0)	2 (1.11)	0.512^a^
Housewife	2 (0.65)	0 (0)	2 (1.11)	0.512^a^
Total	310**	129 (41.75)	180 (58.25)	< 0.001

*NS* nonsignificant.

*Calculated for the total study population (n = 3,863).

**Gestational age was not available for one participant.

^a^Fisher's exact test.

^b^Wilcoxon rank-sum test.

Maternal GBS colonization was tested in women between 18 and 54 years old (mean 30.49 ± 6.44 years). The preterm birth and stillbirth rates were 1.94% and 3.87%, respectively. Statistically significant differences were found between the two gestational age groups with respect to preterm birth (*p* = 0.043), stillbirth (*p* = 0.036), primiparity, in vitro fertilization and gestational age (*p* < 0.001). However, the mean age of pregnant women in the ≥ 35 weeks gestation group and < 35 weeks gestation group was not significantly different (*p* = 0.15). The same result was found for the second parity (*p* = 0.329), the third parity (*p* = 1), the fourth parity (*p* = 0.53) and the 5th parity (*p* = 0.42).

Most of the women with maternal GBS colonization were self-employed (103, 33.23%), followed by office workers (77, 24.84%) and teachers (62, 20%). No statistically significant differences were found in the occupation between the two groups by gestational age (*p* > 0.05).

### Antimicrobial resistance

Table 2 provides the antibiotic-resistant rate of maternal GBS colonization. Among the 310 GBS colonization cases, 272 were tested for antibiotic susceptibility (AST), accounting for 87.74% (95% CI: 83.44–91.08%). All isolates were sensitive to ampicillin, penicillin, ceftriaxone, cefotaxime, vancomycin, and quinupristin/dalfopristin. Resistance to tetracycline was the highest at 89.66% (234/261), followed by erythromycin at 76.23% (202/265), clindamycin at 58.21% (156/268), chloramphenicol at 52.38% (22/42), and levofloxacin at 28.46% (74/260). No statistically significant differences were found for antibiotic resistance rates between the two maternal GBS colonization groups (*p* > 0.05).

**Table 2:** Antibiotic-resistant rate of maternal GBS colonization.

Group	Antibiotics	All isolates (n = 272) %, (R/n*)	≥ 35 weeks (n = 113) %, (R/n*)	< 35 weeks (n = 159) %, (R/n*)	*p* value
Penicillins	Ampicillin 10 µg	0 (0/245)	0 (0/102)	0 (0/143)	–
	Penicillin 10 units	0 (0/243)	0 (0/102)	0 (0/141)	–
Cephems	Ceftriaxone 30 µg	0 (0/33)	0 (0/11)	0 (0/22)	–
	Cefotaxime 30 µg	0 (0/36)	0 (0/14)	0 (0/22)	–
Glycopeptides	Vancomycin 30 µg	0 (0/244)	0 (0/106)	0 (0/138)	–
Streptogramins	Quinupristin/dalfopristin 15 µg	0 (0/207)	0 (0/86)	0 (0/121)	–
Tetracyclines	Tetracycline 30 µg	89.66 (234/261)	91.18 (93/102)	88.68 (141/159)	0.661
Macrolides	Erythromycin 15 µg	76.23 (202/265)	74.53 (79/106)	77.36 (123/159)	0.702
Lincosamides	Clindamycin 2 µg	58.21 (156/268)	57.80 (63/109)	58.49 (93/159)	1
Phenicols	Chloramphenicol 30 µg	52.38 (22/42)	61.54 (8/13)	48.28 (14/29)	0.644
Fluoroquinolones	Levofloxacin 5 µg	28.46 (74/260)	29.70 (30/101)	27.67 (44/159)	0.832
MDR	Multidrug-resistance	60.66 (165^a^/272)	52.21% (59^a^/113)	66.67% (106^a^/159)	0.023

(–) Not examined, *MDR* multidrug resistance (resistant to at least one antibiotic in ≥ 3 drug classes), *R* Number of resistant isolates.

*The number of isolates tested (not all isolates tested are equivalent as some data were missing).

^a^Number of multidrug resistance isolates.

Multidrug resistance (MDR) was found in 60.66% (165/272), and 8.82% (24/272) of isolates were resistant to six to seven antibiotics tested. We found a statistically significant difference in the MDR rate between the two maternal GBS colonization groups (*p* = 0.023).

**Figure 1:**
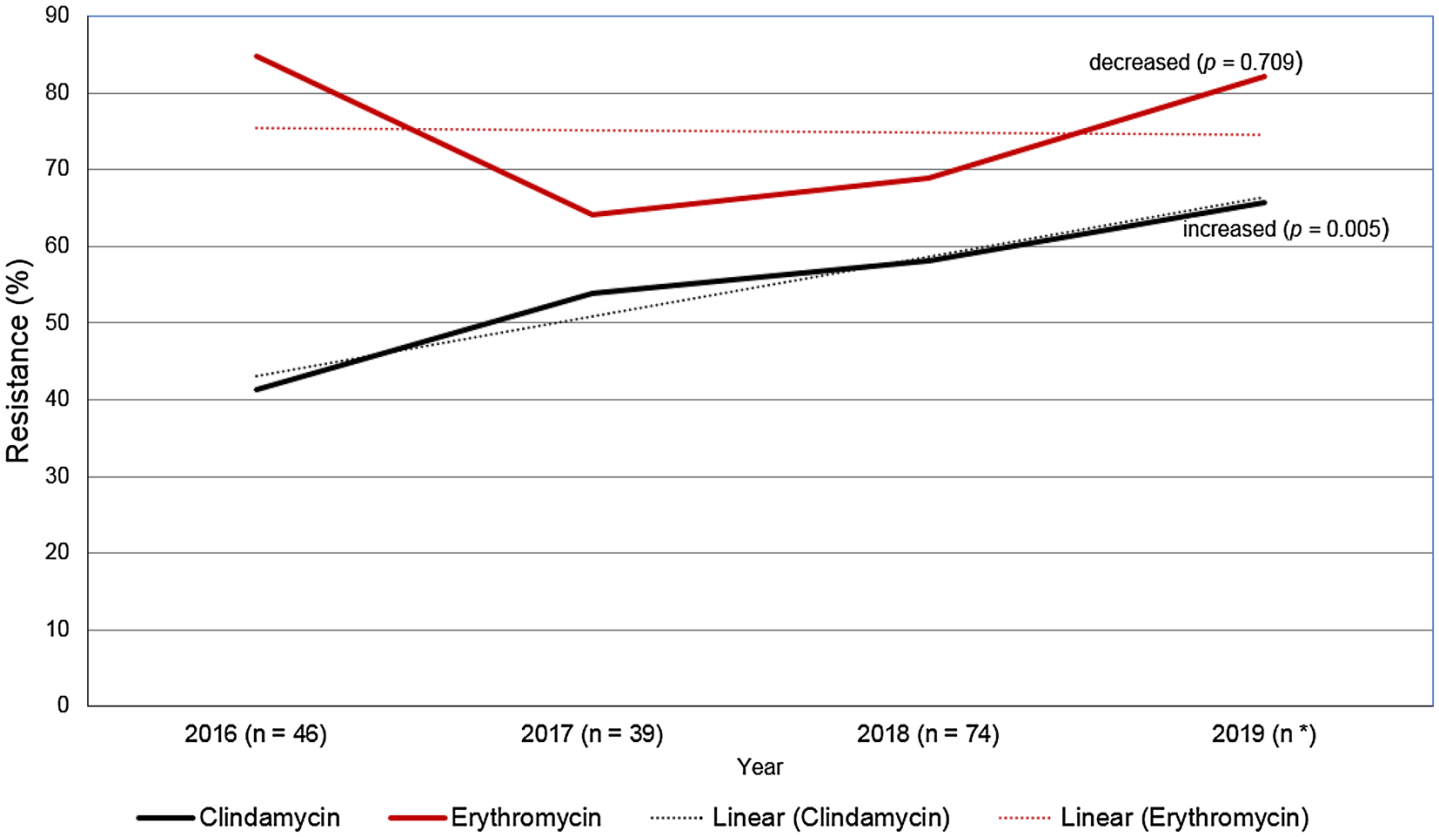
Clindamycin and erythromycin resistance rates among Group B *Streptococcus* isolates by year. *Clindamycin (n = 102) and erythromycin (n = 101); in 2020, due to the COVID-19 pandemic in Vietnam, only 12 GBS strains (< 30) were collected; thus, the clindamycin and erythromycin resistance rates were not calculated.

Clindamycin-resistant but erythromycin-sensitive (L phenotype) was found in 2.2% (6/272). Clindamycin resistance significantly increased from 41.3% of isolates in 2016 to 65.69% in 2019 (*p* for trend = 0.005). Erythromycin resistance decreased from 84.78% of isolates in 2016 to 82.18% in 2019, but the difference was not statistically significant (*p* for trend = 0.709) (Fig. 1).

## Discussion

To date, the rates of GBS colonization and antibiotic resistance in many countries, including Vietnam, are not known. Due to the potential for mother-to-child transmission, screening for GBS in pregnant women is necessary. These data are essential for future GBS interventions. The rate of maternal GBS colonization in this study was 8.02%, which was comparable with India (7.8%)^18^, Korea (9.09%)^8^, and Nghe An, Vietnam (9.2%)^17^. Our result is high compared to the reported rate of maternal SGB colonization in China (3.5%)^19^ and Japan (5.7%)^20^. However, it was low compared to Serbia (15.6%)^21^, Brazil (17.2%)^22^, and Indonesia (30%)^23^. The difference in the rate of GBS colonization can be explained by differences in the region, ethnicity, socioeconomic status, methodological questions, sampling site, laboratory culture techniques, mother’s age, and gestational age^6,8,24^. International standardized sampling for maternal GBS colonization has not been established^6^. The use of standard sampling, detection, and interpreting procedures may contribute to differences in the GBS incidence. We recommend that GBS screening protocols be considered preventative strategies for pregnant Vietnamese women.

In a systematic review and meta-analysis, Russell et al. reported that among studies reporting the GBS colonization prevalence, 82 (26%) were sampled at delivery, and 94 (30%) were sampled from women < 35 weeks of gestation^6^. In a systematic review carried out recently, Abbasalizadeh et al. reported that the prevalence of GBS in pregnant women sampled before the 35th week was greater than that in women sampled after the 35th week^25^. In this context, we separated Vietnamese maternal GBS colonization into two groups, i.e., ≥ 35 versus < 35 weeks of gestation to investigate the differences in the GBS colonization and antibiotic resistance rates between them.

Our results showed that the maternal GBS colonization rate sampled < 35 weeks (4.66%) was higher than that of the ≥ 35 weeks of gestation sampled (3.34%) and the difference was statistically significant (*p* = 0.004). This result is consistent with some studies^6,26^ but inconsistent with others^27^. This variation may be due to differences in demographics, sexual activity during pregnancy, douching habits, and the transient nature of colonization^26,28^. These factors may alter the vaginal microbiota or disturb the vaginal mucosa to promote colonization of GBS. These findings require confirmation in the future.

Penicillin and ampicillin are the first-choice drugs for the prevention and treatment of maternal GBS infections^7^; however, reduced sensitivity to penicillin and ampicillin is becoming increasingly common^11–14^. In this study, all GBSs were susceptible to penicillin, ampicillin, and vancomycin. Thus, these antibiotics would be appropriate for the prophylaxis and treatment of maternal GBS colonization in this study area. Macrolides and lincosamides share similar binding sites, so resistance to one class often crosses the others^29^. Thus, GBS resistance to clindamycin but not to erythromycin (L phenotype) is rare. However, the L phenotype has been documented^2^. In this study, the rate of the L phenotype was 2.2%, which was higher than that in a previous study (0.31%)^2^. This finding suggests that laboratories should consider examining the L phenotype. To our knowledge, this is the first report on the L phenotype among GBS strains of pregnant Vietnamese women.

Erythromycin and clindamycin are the second-choice drugs for colonization of GBS in women allergic to penicillin^7^. However, the antibiotic resistance rate remains high and has increased in recent years. Resistance to other classes of antibiotics (fluoroquinolone and tetracycline) continues to increase, which will cause clinical problems. If patients are allergic to penicillin and the second-choice drugs are not effective, the antibiotic of last resort, vancomycin, is used. However, resistance to vancomycin within GBS has been reported^15^. The rate of resistance to vancomycin in GBS is low, possibly due to the lack of testing for vancomycin susceptibility. In addition, resistance to vancomycin may increase, and this possibility is a concern. Therefore, vancomycin resistance in GBS requires monitoring.

In this study, 76.23% and 58.21% of GBS strains were resistant to erythromycin and clindamycin, respectively. These resistance rates were higher than that of previous studies, e.g., 26.7% and 22.1%^21^, 36.8%, and 7.7%^30^, 50.7%, and 38.4%^31^, indicating that more pregnant women in Vietnam are at risk. High rates of erythromycin and clindamycin resistance threaten the health care of patients, limiting the options to treat mothers and newborns who are colonized with GBS. Further steps are thus required to protect the mother and newborn. These data suggest that clindamycin and erythromycin may be ineffective in treating GBS infections in Vietnamese pregnant women. Therefore, using clindamycin and erythromycin as empirical antimicrobial therapy may be prudent. The high rate of resistance to clindamycin and erythromycin is alarming and requires further investigation.

In pregnant women with penicillin allergies, erythromycin and clindamycin resistance should be tested, and only clindamycin should be reported^32^. The results from this study demonstrate that clindamycin resistance has increased over time (*p* for trend = 0.005), which is consistent with a previous study^33^. Therefore, the choice of an alternative prophylactic antibiotic for patients with penicillin allergies should be based on antibiotic resistance profiles in each region.

The GBS resistance to different antibiotic classes in this study is worrying and indicates that alternative treatment strategies are warranted sooner than later. The antibiotic resistance rates were tetracycline (89.66%), chloramphenicol (52.38%), and levofloxacin (28.46%). The resistance to tetracycline, chloramphenicol, and levofloxacin was high compared with previous studies^34–36^. Monitoring the levels of antibiotic resistance is therefore important. High rates of multidrug resistance (60.66%) and 8.82% resistance to 6–7 antimicrobials tested suggest that antibiotic susceptibility should be determined. The high rate of MDR in GBS is alarming and justifies further investigations.

Due to the lack of resources, there are several limitations to this research. First, the serotype distribution of isolates was not determined. As such, data on the serotype distribution of GBS in Vietnam remains insufficient for vaccine development. Second, although GBS genotypic analysis is useful for AST verification, it could not be conducted due to limited resources. As the GBS strains were not cryopreserved, retrospective genotypic analyses were impossible to perform. However, the rate of antibiotic resistance highlights the present problem in Vietnam and raises important questions for access to molecular methods in low- and middle-income countries. Third, the frequency of mother-to-newborn transmission of GBS was not estimated, and risk factors for both outcomes were not identified. Only detailed information on maternal GBS colonization was collected; thus, the baseline characteristics of mothers who were not colonized with GBS were not fully elucidated. We carried out convenience sampling in one hospital, so the results may not represent all pregnant women in Vietnam.

Despite these limitations, this is the first study in Vietnam over 5 years to show the maternal colonization and antibiotic resistance profiles of GBS. A larger multisite study on neonatal transmission, serotype distribution, and molecular characterization of GBS is required.

## Conclusion

The GBS colonization rate in this study was low and within the range of other global studies. The resistance to erythromycin and clindamycin and the multidrug resistance rate were high. Clindamycin resistance increased significantly throughout the study period. Penicillin and ampicillin are the drugs of choice for preventing and treating GBS-related maternal infections in the study area. Ongoing monitoring of newborn transmission, serotype distribution, and molecular characterization of GBS should be considered in the future.

## Materials and methods

### Ethics

This study was approved by the Institutional Review Board of Vietnam Military Medical University, Hanoi, Vietnam (VMMU-IEC-AMR-03–20151605-V3). All methods were carried out in compliance with the Declaration of Helsinki. Written informed consent was obtained from all study participants.

### Study area

This study was conducted at the National Hospital of Obstetrics and Gynaecology (NHOG) in Hanoi, Vietnam, between January 2016 and December 2020. NHOG is the largest reference hospital in northern Vietnam, with approximately 1500 beds. The hospital provides primary and tertiary care to almost all pregnant women in northern Vietnam and treats pregnant women with associated diseases.

### Study design

This observational study was conducted from January 2016 to December 2020 at NHOG, Vietnam. Pregnant women who attended the National Hospital of Obstetrics and Gynaecology (NHOG) in Hanoi, Vietnam, were enrolled. Pregnant women were included in the study if they had not used antibiotics in the last 3 weeks and provided informed consent.

### Specimen collection, handling, and transport

Vaginal-rectal swabs were collected from pregnant women before vaginal examinations and as described previously^24^. Briefly, a single flocked swab (BD Diagnostics, Korea) was used to collect the vaginal-rectal specimens. First, the swab was inserted ~ 2 cm without the use of a speculum into the vagina, and then the same swab was inserted ~ 1 cm through the anal sphincter. After specimen collection, the swabs were immediately inserted into Amies transport medium (Thermo Scientific™, Singapore) and transported to the Medical Microbiology Department at NHOG within 4 h.

### GBS identification

The samples were processed as recommended by the American Society for Microbiology’s guidelines^24^. Briefly, after incubating the swabs in Todd-Hewitt broth (Thermo Scientific™, Singapore) aerobically at 37 °C for 18–24 h, 10 μl of each broth was subcultured on Columbia agar plates with 5% sheep blood (Oxoid, Singapore). The plates were incubated for 24 h at 37 °C in 5% CO_2_. If GBS was not detected, the blood agar plate was incubated and examined after 48 h. All suspected GBS appeared as either beta-haemolytic or nonhemolytic, and Gram-positive cocci and catalase-negative cocci were taken for the CAMP (Christie–Atkins–Munch-Peterson) test. *Streptococcus pyogenes* ATCC 19615, *Streptococcus agalactiae* ATCC12386, and *Staphylococcus aureus* ATCC 25923 (Thermo Scientific™, Singapore) were the controls. All colonies that yielded a positive CAMP were considered GBS. Positive CAMP test results were confirmed using the Streptex™ Latex Agglutination test (Thermo Scientific™, Singapore) according to the manufacturer’s instructions. This latex agglutination test provides a complete solution for the isolation and differentiation of Lancefield Groups A, B, C, D, F, and G.

### Antimicrobial susceptibility testing

The antimicrobial resistance of GBS was determined through the disk diffusion method (Kirby-Bauer), Vitek 2 using the AST-ST03 card (bioMérieux, France), or E-test (bioMérieux, France). The clinical isolates were tested for susceptibility to 11 different antibiotics, including ampicillin 10 µg, penicillin 10 units, cefotaxime 30 µg, ceftriaxone 30 µg, vancomycin 30 µg, quinupristin/dalfopristin 15 µg, tetracycline 30 µg, erythromycin 15 µg, clindamycin 2 µg, chloramphenicol 30 µg, and levofloxacin 5 µg (Oxoid, Singapore). *Streptococcus pneumoniae* ATCC 49619 was the control.

The GBS colonies from an overnight (18 to 20 h) sheep blood agar plate were suspended in 5 ml of sterile saline, and the turbidity was adjusted to 0.5 McFarland using the DensiCHEK Plus instrument (bioMérieux, France). The GBS suspension was streaked over the dried surface of 5% sheep blood Mueller–Hinton agar plates (bioMérieux, France) using a sterile swab. The antibiotic disks/or E-tests were placed on the plates and incubated for 20–24 h at 37 °C in 5% CO_2_. After incubation, the inhibition zone around the disks/or E-test was analysed. Vitek 2 susceptibility testing was performed according to the manufacturer’s instructions with the same bacterial suspension using the AST-ST03 card. The results were analysed and interpreted by AES 4.02 software. The isolates were considered susceptible, resistant, or intermediate according to the recommendations by the CLSI (Clinical and Laboratory Standards Institute)^32^. Multidrug resistance was defined as resistance to at least three antibiotics in at least three antimicrobial classes.

### Data analysis

Maternal GBS colonization was calculated for the entire study population. All data were separated by gestational age group (35 vs. < 35 weeks). The results of antibiotic resistance indicated the percentage of isolates tested resistant to certain antimicrobials for each identified phenotype. The clindamycin and erythromycin resistance percentages each year were only calculated when the number of isolates was ≥ 30 to ensure a minimum level of precision in the calculation.

### Statistical analysis

Statistical analyses were performed using R 4.0.5^37^. The chi-square or Fisher's exact test was used to compare categorical variables; Fisher's exact test was used for variables with less than five in at least one cell. The chi-square test for trend was used to examine trends across the study period. For continuous variables, the Kolmogorov–Smirnov test or the Shapiro–Wilk test (depending on the data number) was used to test for normality; the Wilcoxon rank-sum test (two-tailed) was used for nonnormally distributed data; Student’s *t*-test (two-tailed) was used for normally distributed data. A *p value* below 0.05 was considered statistically significant.

## Data Availability

The data supporting the reported results are available on request.
